# Access block and overcrowding at the emergency department at Tupua Tamasese Meaole Hospital in Samoa

**DOI:** 10.1186/s12245-023-00512-1

**Published:** 2023-05-08

**Authors:** Tamara Ah Leong-Nowell, Ledua Tamani, Annette Kaspar

**Affiliations:** 1 School of Public Health and Primary Care, Fiji National University, Suva, Fiji; 2 Samoa Medical Association, Apia, Samoa; 3 Tupua Tamasese Meaole Hospital, Ministry of Health, Apia, Samoa

**Keywords:** Access block, Overcrowding, Emergency department, Pacific Islands

## Abstract

**Background:**

Access block and overcrowding are known to adversely impact on patient outcomes, service delivery, and patient experiences within emergency departments (ED) worldwide. There are no studies on access block or overcrowding from the Pacific Islands. The aim of the present study is to provide preliminary data on access block and overcrowding in the ED of the national tertiary hospital of Samoa.

**Methods:**

Mixed methods study design. Data collection was performed in March 2020. The quantitative strand calculated (1) the point prevalence of patients impacted by access block in the ED, and (2) the ED bed occupancy rate to assess for overcrowding. The qualitative strand used thematic analysis of two focus group interviews exploring access block and overcrowding with ED medical and nursing staff members.

**Results:**

On the day of data collection, a total of 60 patients presented through the ED triage system. Of the 20 patients who were admitted into ED, 80% were triaged as ‘see without delay’ (CAT1), ‘emergency’ (CAT2) or ‘urgent’ (CAT3). For patients requiring admission to hospital wards, 100% waited 4 + h in ED, and 100% waited 8 + h, suggesting the presence of access block. Overcrowding in the ED setting was also evident, with an ED bed occupancy rate of 0.95, and an adjusted bed occupancy rate of 1.43. The major themes emerging from the ED staff focus groups and individual in-depth interviews were (1) the adverse impacts of access block and overcrowding, i.e., violence towards ED staff members, (2) the preventable contributing factors, i.e., lack of physical beds in the ED, and (3) practical recommendations to improve patient flow through the ED, i.e., improved collaboration between ED, outpatient services, and the hospital wards.

**Conclusions:**

Preliminary evidence suggested the presence of access block and overcrowding in the ED of the national tertiary hospital of Samoa. ED staff interviews provided insight into the ED frontline challenges and offered practical recommendations for ED health service improvement.

## Background

Access block and overcrowding in hospital emergency departments (EDs) is a major public health issue impacting on patient outcomes and healthcare services worldwide [1, 2]. The well-known adverse effects of access block and overcrowding are wide-ranging, and include delays in critical emergency care, preventable patient mortality, and violence towards emergency staff members [3–5]. Given that these concerns may be addressed through improvements in health care system delivery, access block and overcrowding have become an essential component of the literature on emergency medicine research [6–10]. As the world adjusts to the aftermath of the COVID-19 pandemic, a review of emergency health services and their delivery is timely.

The International Federation of Emergency Medicine describes access block as the time a patient spends in the ED between their hospital admission and inpatient bed availability [1, 2]. Access block definitions vary internationally, from 4 h or more, to 8 h or more, time spent in the ED. Overcrowding refers to the situation where clinical care demand outweighs clinical care supply. Although there are a number of contributing factors, a bed occupancy rate of greater than1.0 is routinely used as an overall indicator of overcrowding [11].

While there is a wealth of research from high-income nations on access block and overcrowding, a review of the literature revealed that there is very little evidence from low- and middle-income countries. A multicentre study from Nigeria found that overcrowding and long waiting times in the ED were the leading causes of violence towards staff members, with 65% of staff reporting that they were the direct victims of violence [12]. The violence was mostly perpetrated by visitors to the ED, and nurses were significantly more affected than medical officers [12]. A second study from Thailand reported that the main reason for waiting times in ED was due to multiple rounds of blood testing for differential diagnosis [13]. A final study from Malaysia reported a significant reduction in access block prevalence when internal medical specialists personally attended the patient in the ED setting to offer timely diagnosis and intervention [14]. There are no research studies on access block or overcrowding from the Pacific Islands.

**Fig. 1:**
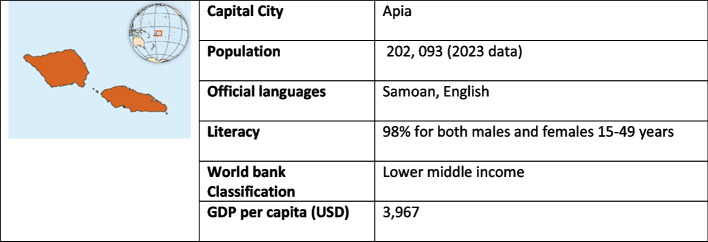
Samoa country profile

The aim of the present study was to determine if access block and overcrowding exist in the main referral hospital of Samoa, a Polynesian nation of the Pacific Islands (Fig. 1). Tupua Tamasese Meaole (TTM) Hospital is located in the capital Apia, and is the major centre for national emergency health services. Patients presenting to the ED are routinely evaluated by the ED triaging nurses, who may admit them to the ED, direct them to the most appropriate hospital outpatient service, or discharge them with advice on home care (i.e., positive COVID-19 screening test result). With a total of 21 licenced treatment bays, it is the only national emergency setting that is staffed by medical officers with post-graduate qualifications in emergency medicine. Should there be evidence of access block or overcrowding, a second aim of the study will be to determine the contributing factors in order to inform health service delivery quality improvement. This aligns with the Health Sector Plan mission statement of “Enhancing public health to provide people-centred health services”, as well as the national health goals under the United Nations Sustainable Development Agenda. The results of the study should be translational, and prove useful to both our Pacific Island neighbours and other low- and middle-income country settings.

## Methods

Ethical approval for the study was obtained from (1) the College of Health Research and Ethics Committee (CHREC) at the Fiji National University, and (2) the Health Research Ethics Committee of the Government of Samoa Ministry of Health. Gatekeeper approval was also obtained from the director of TTM Hospital and the Head of the Emergency Department.

The study was conducted at the ED of TTM Hospital towards a Masters research qualification. A mixed methods approach was selected, with a convergent parallel design using convergent triangulation.

### Quantitative strand

The quantitative strand of the study used a convenience sampling method to evaluate the characteristics of patients who accessed the TTM Hospital ED on the days of data collection. The methodology for point prevalence calculation was modelled on a study from New Zealand [15], where 2 days of collection were selected for comparisons. Based on the methodology of an Australian study investigating access block [16], a modified version of their data collection sheet was developed to record the required information of patients seen over the 24-h period on the selected days of data collection. The following de-identified data were extracted from the emergency department triage registry books and the emergency department admission record books: age (years), gender (male, female), time of presentation to ED (AM Shift [0800–1600], PM Shift [1600–2400], night shift [2400–0800), presenting complaint, disease category (CAT1:to be seen without delay, CAT2:emergency:10 min, CAT3:urgent:30 min, CAT4:semi-urgent:60 min, CAT5, CAT6), ED Triage Plan (discharge home, refer to ED, refer to another clinic, patient left without being seen), diagnosis, ED outcome (refer to paediatrics ward, refer to medical ward, refer to surgical ward, discharge home, no plan/incomplete record, patient died in ED), and time spent in ED (< 1 h, 2 h, 3 h, 4 h, 5 h, 6 h, 7 h, 8 + h).

Data were entered into a purposefully designed Excel spreadsheet, and descriptive statistics were performed by the chief investigator (TALN) in collaboration with the Fiji National University statistician. Access block was determined by calculating the proportion of patients who were admitted to a hospital ward but kept in ED for 4 + or 8 + h. Occupancy rate was determined by calculating the proportion of total number of admitted patients in ED against the total number of licensed treatment bays (excluding hallways) over the 24-h data collection period. We note that our methodology of bed occupancy rate calculation aligns with inpatient unit practice, rather than that which is more commonly used by emergency departments internationally [17].

### Qualitative strand

The qualitative strand of the study used focus groups and in-depth interviews to explore the perspectives of frontline ED health workers on access block and overcrowding. Study population eligibility and inclusion criteria were all nurses (*N* = 19) and doctors (*N* = 5) employed by ED TTM Hospital at the time of the study data collection. Information was provided about the study, and all willing participants signed a consent form prior to data collection.

The focus groups and in-depth interviews were facilitated by the chief investigator (TALN), and conducted in English and/or Samoan depending on participant preferences. The focus groups consisted of junior nursing staff members, while the in-depth interviews consisted of medical officers and senior nursing staff members. The following questions were used to prompt discussion regarding access block and overcrowding:

1. What do you know of access block, bed block, ED overcrowding?
2. Do you think that access block is a problem in the ED of TTM Hospital?
3. What are some of the factors that cause access block?
4. Is ED overcrowding a problem in TTM Hospital?
5. What contributes to ED overcrowding?
6. What are some of the problems noted when there is overcrowding in the ED?
7. What are some solutions to access block?
8. What are some solutions to ED overcrowding?

The discussions were recorded, and then transcribed into English by the chief investigator (TALN). Thematic analysis was again performed in collaboration with the Fiji National University statistician. The most common themes were identified, and then further classified as major themes with corresponding sub-themes.

## Results

### Quantitative strand

Data collection was impacted by the outbreak of the COVID-19 pandemic when Samoa implemented a State of Emergency in April 2020. Given the time restrictions imposed for completion of the Masters research program, the study data collection was limited to one day only (Monday 7am 16th March to Tuesday 7am 17th March).

A total of 60 patients accessed the ED Triage System on the day of data collection. There were 15 males (25%), 31 females (51.7%), and 14 cases with missing gender information (23.3%). The age distribution was 0–20 years (*n* = 18, 30%), 21–40 years (*n* = 8, 13.3%), 41–60 years (*n* = 6, 10%), 61–80 (*n* = 10, 16.7%), 81–100 years (*n* = 3, 5%), and 15 case were missing data (25%). The distribution according to severity of condition was CAT1 (*n* = 3, 5%), CAT2 (*n* = 6, 10%), CAT3 (*n* = 20, 33.3%), CAT4 (*n* = 17, 28%), and missing data for 14 (23.3%) of cases. The most common presenting complaints to ED Triage were flu-like symptoms (25%), acute abdominal pain (15%), and musculoskeletal (13.3%), and constitutional symptoms (13.3%).

A total of 20 patients were admitted from Triage to the emergency department on the day of data collection. There was an equal number of male and female patients. The distribution according to age categories was 0–20 years (*n* = 2, 10%), 21–40 years (*n* = 2, 10%), 41–60 years (*n* = 2, 10%), 61–80 years (*n* = 10, 50%), 81–100 years (*n* = 3, 15%), and 1 case with missing data (5%). The AM Shift and night shift both recorded 40% of admissions, with the PM Shift accounting for the remaining 20%.

The most common diagnoses were sepsis (24.4%), followed by acute illness (22.2%) (i.e., acute allergic reaction, food poisoning, arrhythmias, stroke, seizures, fluid overload), followed by trauma/MVA (17.8%). A total of 7 (35%) cases were successfully managed by the ED and discharged home. A total of 8 (40%) of cases were referred to the appropriate hospital ward: medical (25%), paediatrics (10%), and surgical (5%). One case died in the ED, and records were incomplete for the four remaining cases.

The access block point prevalence calculations are summarised in Table 1. Of the 8 patients requiring transfer to a hospital ward, all 8 patients spent 8 + h in the ED, which computes to an access block point prevalence of 100%. Of the 8 patients requiring transfer to a hospital ward, 8 patients spent 4 + h in the ED, which computes to an access block point prevalence of 100%.

**Table 1:** Total hours in emergency department and point prevalence access block (*N* = 8)

Patient	Ward admission	Time in ED (hours)	4 + h in ED awaiting transfer to hospital ward	8 + h in ED awaiting transfer to hospital ward
1	Paediatrics	8	Yes	Yes
2	Paediatrics	8	Yes	Yes
3	Surgical	13	Yes	Yes
4	Medical	9	Yes	Yes
5	Medical	8	Yes	Yes
6	Medical	13	Yes	Yes
7	Medical	8	Yes	Yes
8	Medical	20	Yes	Yes
Point prevalence access block			100%	100%

There are 21 beds in the emergency department. Over the 24-h data collection period, there was a total of 20 admissions, corresponding to a bed occupancy rate of 0.95. Given that only 14 ED beds were available on the day of data collection since 7 ED beds had previously accompanied patients to the hospital ward to which they were admitted (N.B. these are separate from the 8 ED beds described in Table 1), the adjusted bed occupancy rate for this 24-h period was 1.43.

### Qualitative strand

Again, due to the outbreak of the COVID-19 pandemic and the time restrictions imposed for completion of the Masters research project, the focus groups and in-depth interviews were conducted during the month of March 2020 only.

Overall, 17 of the 24 eligible ED staff members (70.8%) took part in the study. Non-participants were on leave on the day of data collection. There were two focus group discussions conducted with twelve nurses employed at the emergency department: seven nurses had worked in the ED between 1 and 2 years, and the remaining 5 nurses had over 5-year ED experience. The in-depth interviews were conducted with three ED medical officers and two senior nurses with 19–24-year experience in the ED (N.B. these two senior nurses are separate from and in addition to the nurses participating in the focus groups). An overview of the major themes and sub-themes are summarised in Table 2.

**Table 2:** Overview of thematic analysis results for focus group discussions and in-depth interviews

Theme	Sub-theme
Adverse impacts of access block and overcrowding in the ED	Emotionally disturbed patients and accompanying caregivers
	Violence towards ED staff members
Contributing factors to access block and overcrowding in the ED	Transfers from the outpatient clinic when it closes at 10 pm
	Only one medical officer on duty
	Different practices between senior and junior medical officers
	Delayed laboratory test results
	No physical bed to accommodate patients
	Bed unavailability on the hospital ward
	Delays in the patient discharge process from the hospital ward
Practical solutions to access block and overcrowding in the ED	Use of patient care co-ordinators
	Improved assessments of patients who may be discharged to homecare and reviewed in the outpatient clinics
	Use of discharge lounge on the wards to improve bed availability and turnover times
	Investment in acute critical care training
	Improved working relationships between the ED, outpatient clinics, and hospital wards

The first theme to emerge was the adverse impacts of access block. Given that presentation to the emergency department is an inherently stressful situation for both patients and their attending caregivers, delays in care due to access block will often exacerbate an already emotionally charged environment. Visitors to the ED may turn violent towards hospital staff. Participants reported that while some people are understanding when the triage system is explained to them, others will continue to complain and cause a negative atmosphere within the emergency department setting.

“There are different kinds of people, there are some who will understand when we explain the delay but there are some who just look like they are ready to come at you.” Participant, Focus Group

“..some people, you can just hear them complaining out loud.” Participant, Focus Group

“Sometimes people get angry because we have to explain that according to the triage system patients are categorised according to the severity of the illness they present with, and that there is a time frame…however when there is an emergency and the only doctor is called to tend to this emergency, sometimes that time frame is not kept.” Participant, In-depth Interview

The second theme to arise was the participant perspectives on various contributing factors to access block and overcrowding in the ED. The Acute Patient Care Clinic (APCC) is a hospital outpatient service which closes at 10 pm, and participants reported that there may be no alternatives for people under observation in APCC other than transfer to the ED. This issue is compounded by a lack of bed availability.

“when the nurses in APCC hand over their patients for admission to ED sometimes there are not beds available.” Participant, In-Depth Interview

Participants also reported challenges associated with the availability of medical officers, and the different work practices between junior and senior physicians.

“Having one doctor on the floor (to work in ED) is another problem that we face, and we always get abused by people because they keep asking ‘why is there a delay in sending us to the ward?’…we have to wait until ward rounds are done, but you can see the patient is deteriorating…the abuse continues because there is a delay in seeing these patients”. Participant, In-Depth Interview

“When we call to say that the results are not ready but that the on-call doctor needs the patient to be admitted, the senior doctors will admit, they will not wait for the results. But the juniors, they wait for the results even if the patients are very sick…everything goes downhill for us.” Participant, Focus Group

Another recurring sub-theme was that delays in laboratory investigations contributed to ED waiting times. The adverse impact is particularly evident when the attending physicians prefer laboratory confirmation prior to any decision-making on hospital admissions or treatment plans.

“the first thing is the slow processing of results for patients from the lab…unless the results come, only then will patients be referred…if we have too many cases then we sometimes refer straight away…but then again, it depends on who the on-call doctor is.” Participant, Focus Group

“The lab is another big problem here, sometimes we are at the end of our shift but there are still no results for patients”. Participant, Focus Group

A final subtheme of contributing factors to ED access block and overcrowding was related to hospital ward service provision. The hospital wards are sometimes unable to receive transfers from ED due to their own bed availability constraints, which may be confounded by delays in the patient discharge process.

“One of the main factors that really affects access block is probably the capacity of the wards in terms of patient load…I think that is probably the most important factor of access block in the hospital”. Participant, In-Depth Interview

“We call upon the wards to find out why there is a delay in discharging patients so we can move the boarders down. We are told that the ward rounds have not yet been completed, or the doctor has not arrived to fill in the discharge summary and prescription”. Participant, In-Depth Interview

Study participants also expressed the opinion that hospital ward colleagues were not always truthful about bed availability, leading not only to access block and overcrowding, but also to strained professional relationships.

“Sometimes they even lie saying there are no available beds, that’s why we follow up with the doctors to discharge patients.” Participant, In-Depth interview

The third theme to arise from the focus groups and in-depth interviews was recommendations for effective solutions to access block and overcrowding in the ED. The Patient Care Co-Ordinator (PCC) role was seen as very valuable.

“The PCC are often used, mostly after-hours. They get informed about patients and how long they have been kept in ED for… the PCC is very useful in situations like this i.e. clearing patients down to the wards for admission.” Participant, Focus Group

Improving the discharge process was also seen as a key contributor to reducing access block.

“The one thing that usually helps clear out patients is for those who have been monitored and are improving – they are discharged home with prescriptions and a review appointment in a few days’ time.” Participant, Focus Group

The use of discharge lounges on hospital wards was suggested as an option to improve turnover time and bed availability:

“I remember they used to have a discharge lounge in the medical and surgical wards…in ED, once we discharge our patients we put them in wheelchairs and take them to the triage area to wait for their car…” Participant, In-Depth Interview

As well as investment in critical care training, it was suggested that patient outcomes may be improved if senior physicians tend to critical patients.

“I think one of the most important factors is investing in training. If we invest in training, you have the knowledge of what acute critical care is…you identify who gets to go home and the time they should be sent home.” Participant, In-Depth Interview

“One of the senior doctors should attend to the very sick patients…if the patient is not too critical, a junior doctor can admit the patient. This will relieve the pressure and workload in ED.” Participant, Focus Group Strengthening the working partnerships between APCC and hospital ward should also make a significant contribution to patient care and outcomes.

“APCC should be open 24 hours. Both services -outpatient and ED – should work together. APCC usually has three or four doctors working a shift while ED usually has one.” Participant, Focus Group

“After it closes at 11pm, it would be nice for those doctors (APCC) to cover the triage area while the ED doctors focus on ED patients.” Participant, Focus Group

## Discussion

Preliminary data provides evidence of access block and overcrowding in the emergency department of the national hospital of Samoa. It is difficult to compare our results with other studies given that there are major discrepancies between definitions and reporting styles internationally. This should not deter from the key finding, however, that a preliminary baseline has been established for access block and overcrowding in the TTM Hospital of Samoa, and that future studies may use this as a monitoring and evaluation benchmark to assess for improvements in hospital emergency healthcare services.

Violence towards ED health professionals was identified as a major cause of concern in Samoa, similar to the findings from the Nigerian study [12]. A significant proportion of ED nurses in Samoa are young new graduates. Should violence against ED staff remain unaddressed, it will negatively impact on staff health and well-being, leading to increased sick days, absenteeism, high staff turnover, loss of skilled ED health professionals, and significant emergency health staff shortages. The evidence from high-income countries suggests that the COVID-19 pandemic has renewed community respect for frontline health staff [18], and it is in this spirit of goodwill that a review of ED staff health and safety is recommended as part of the evaluation of the national response to the COVID-19 outbreak in Samoa.

Similar to the other studies from low- and middle-income settings, the present study found that waiting for laboratory results and the early involvement of ward physicians were major factors that impacted access block and overcrowding in the ED [13, 14]. A difference between the Samoan and Thai studies was that waiting for laboratory results in Samoa meant awaiting the first set of requested results, while the Thai experience meant awaiting differential diagnosis from multiple rounds of laboratory testing. A difference between the Samoan and Malaysian studies was that while Samoan physicians are routinely actively involved in ED patient care, the competing constraints of their own wards often prevent their timely attendance in ED.

Although the present study focused on the perspectives of the ED medical staff, it is clear that access block and overcrowding are challenges faced throughout the hospital. There is a shortage of medical officers in the wards as well as in ED, and all medical officers are constantly battling competing priorities. The formulation and implementation of clear policies and/or guidelines may be helpful, especially to early career physicians to help them navigate their working environment.

The preliminary data from the study suggests that the largest proportion of ED patients are admitted to the Medical Ward. Therefore, improving the flow of patients from ED to the Medical Ward merits attention as a significant starting point for reducing access block and overcrowding in the ED. The study participants offered suggestions for both ED and the Medical Ward in this regard, for example the presence of a Discharge Lounge to enable timely availability of hospital ward beds.

### Future directions

A larger study is desirable to enable on-going monitoring and evaluation of access block and overcrowding in emergency settings in Samoa. Given that access block and overcrowding are issues with multiple contributing factors, there are a number of points along the patient journey that warrant further investigation. An incidental finding of the present study was that only two cases in the 0–20-year age group were admitted to ED, even though this age group overwhelmingly represented the largest proportion of presentations to ED Triage (30%): a greater understanding of health-seeking behaviour for paediatric cases may contribute to improvements in the efficiency of ED Triage and ED admission.

Another incidental finding was that the highest proportion of people admitted into the ED are in the 61–80-year age group (50%), and that further admission to the Medical Ward is highly likely: strategies that focus on this age group and their presenting conditions should also be prioritised. Anecdotally, a common difficulty is that hospital beds will malfunction under the weight of patients in this older adult age group who often present with non-communicable disease co-morbidities (i.e., overweight/obesity): to optimise patient safety during transfers from the ED to the hospital wards, the procurement of quality beds that support the needs of the patient population would be a worthwhile and cost-effective investment.

The ED has modified its practice considerably since the outbreak of the COVID pandemic. A review of the triage and flow of patients through ED since the outbreak of COVID-19 will be beneficial, not only to evaluate the positive impacts of COVID-response efforts on hospital service delivery, but to also identify areas for on-going service improvement. Although outside the scope of the present study, it was noted that 39.3% of people attending the ED Triage System left without being seen by a physician. This is a situation that was also identified by the International Federation of Emergency Medicine (IFEM) as a major concern worldwide [2], and it warrants further investigation in future studies in Samoa. It was also noted that there was no Pacific Island representation on the IFEM Task Force, and this should be considered to ensure that the unique context of the Pacific Islands is included in future global studies.

### Limitations of the present study

The present study was conducted at the outbreak of the COVID-19 pandemic. While a larger sample of quantitative and qualitative data were desirable, it was not possible given the time limitations for completion of the Masters research program. Although this study presents preliminary data only, when the results were shared with medical colleagues of Samoa during our weekly continuing education session, it was generally agreed that findings reflected the reality of our national ED hospital setting.

The bed occupancy rate should be interpreted with caution. There was no time element applied to the calculation other than the 24-h data collection period, and the 20 ED admissions were not in ED for 24 h each. Given that variance of length of stay is likely to change ED bed occupancy rate, future studies should include this time factor in their calculations. This recommendation further ensures adherence to international guidelines on calculating bed occupancy rates in emergency departments based on “hours” rather than “days”.

The research methodology for the present study did not adhere to any systematic or established guidelines for thematic analysis of qualitative data. Adherence to well-known methods, such as Braun and Clarke [19], would have been beneficial for study robustness, reproducibility, and future monitoring and evaluation studies.

## Conclusions

Preliminary data provides evidence of access block and overcrowding at the ED of the national hospital in Samoa. While frontline staff have identified the adverse effects of access block and overcrowding on patient outcomes and staff health and well-being, they have also provided practical solutions for improvements to emergency healthcare delivery.

## Data Availability

The data may be accessible upon request from the chief investigator T.A.L.N.
